# Large XPF-dependent deletions following misrepair of a DNA double strand break are prevented by the RNA:DNA helicase Senataxin

**DOI:** 10.1038/s41598-018-21806-y

**Published:** 2018-03-01

**Authors:** Julien Brustel, Zuzanna Kozik, Natalia Gromak, Velibor Savic, Steve M. M. Sweet

**Affiliations:** 1 Genome Damage and Stability Centre (GDSC), University of Sussex, Brighton, BN1 9RQ UK; 2 Sir William Dunn School of Pathology, University of Oxford, Oxford, South Parks Road, OX1 3RE UK; 3 Brighton and Sussex Medical School (BSMS), University of Sussex, Brighton, BN1 9RQ UK; 4 Present Address: Horizon Discovery Ltd, 8100 Cambridge Research Park, Cambridge, CB25 9TL UK; 5 Present Address: NantOmics, 9600 Medical Center Drive, Rockville, MD 20850 USA

## Abstract

Deletions and chromosome re-arrangements are common features of cancer cells. We have established a new two-component system reporting on epigenetic silencing or deletion of an actively transcribed gene adjacent to a double-strand break (DSB). Unexpectedly, we find that a targeted DSB results in a minority (<10%) misrepair event of kilobase deletions encompassing the DSB site and transcribed gene. Deletions are reduced upon RNaseH1 over-expression and increased after knockdown of the DNA:RNA helicase Senataxin, implicating a role for DNA:RNA hybrids. We further demonstrate that the majority of these large deletions are dependent on the 3′ flap endonuclease XPF. DNA:RNA hybrids were detected by DNA:RNA immunoprecipitation in our system after DSB generation. These hybrids were reduced by RNaseH1 over-expression and increased by Senataxin knock-down, consistent with a role in deletions. Overall, these data are consistent with DNA:RNA hybrid generation at the site of a DSB, mis-processing of which results in genome instability in the form of large deletions.

## Introduction

DNA is the target of numerous genotoxic attacks that result in different types of damage. DNA double-strand breaks (DSBs) occur at low frequency, compared with single-strand breaks and other forms of DNA damage^1^, however DSBs pose the risk of translocations and deletions and their repair is therefore essential to cell integrity. The majority of DSBs are repaired by either homologous recombination (HR) or non-homologous end-joining (NHEJ), with a smaller fraction repaired by non-canonical alternative end joining and single-strand annealing pathways^2–5^. In order to study the repair of a DSB at a known site in the genome, rare-cutting endonucleases such as I-SceI are employed^6^. DSBs generated by endonucleases have ‘clean’ ends, i.e. intact 5′-phosphate and 3′-hydroxyl groups, and are in most cases repaired without end-processing and associated deletions^7,8^.

R-loops consist of an RNA:DNA hybrid, with the RNA displacing the non-transcribed DNA strand^9^. R-loops are a source of genome instability^9,10^. Indeed, collisions between replication or transcription machineries with R-loops can result in DSBs. It has recently been shown that Fanconi anemia proteins prevent instability resulting from replication fork progression and R-loops^11,12^. Furthermore, the displaced single-stranded DNA resulting from R-loop formation is susceptible to damage or processing. For example it has been shown that the transcription-coupled nucleotide excision repair (TC-NER) pathway, including flap endonucleases XPF/ERCC4 and XPG/ERCC5, can generate DSBs after R-loop formation^13^. Recently it has been demonstrated in *S. pombe* that DNA:RNA hybrids can occur in a DSB-dependent manner, associated with PolII recruitment to the DSB region^14^. These DNA:RNA hybrids are presumed to originate from transcription from the DSB and the displaced DNA strand is either resected or free-floating. DNA damage-dependent DNA:RNA hybrids have also been detected in human cells^15^. Transcription initiated from DSBs in human, *Drosophila* and plant cells has been reported^16–19^.

To prevent the formation of R-loops, RNA-binding proteins interact with the RNA transcript, preventing it from invading the DNA duplex^10^. In parallel, topoisomerase enzymes resolve R-loop-promoting negative supercoiling, generated behind polymerases^10,20^. In addition, the cell possesses two different mechanisms to remove R-loops: the DNA-associated RNA can be specifically digested by enzymes of the RNase H family; the DNA:RNA hybrid can be dissociated by DNA:RNA helicases such as Senataxin, Aquarius and others^13,21,22^. Removing the protective function of Senataxin results in an increase in DNA strand breakage and γH2AX: these effects are reduced with overexpression of RNaseH1, implicating increased R-loops in the damage^23^.

In this report, we have established a new system to study the deleterious consequences of DSBs utilising a proximal transcription unit as a marker. We show that targeted DSB induction and repair is correlated with an appearance of a subpopulation where the neighbouring gene is lost due to a large deletion. Knockdown of the DNA:RNA helicase Senataxin increases deletions, while RNaseH1 over-expression and knockdown of the 3′ flap endonuclease XPF/ERCC4 has the opposite effect. DNA:RNA hybrids were only detected after DSB induction. These results suggest a role of DNA:RNA hybrids in DSB processing, defects in which can result in genome instability in the form of large deletions.

## Results

### A two-component system to study the long-term effect of DNA damage on a neighbouring gene

**Figure 1:**
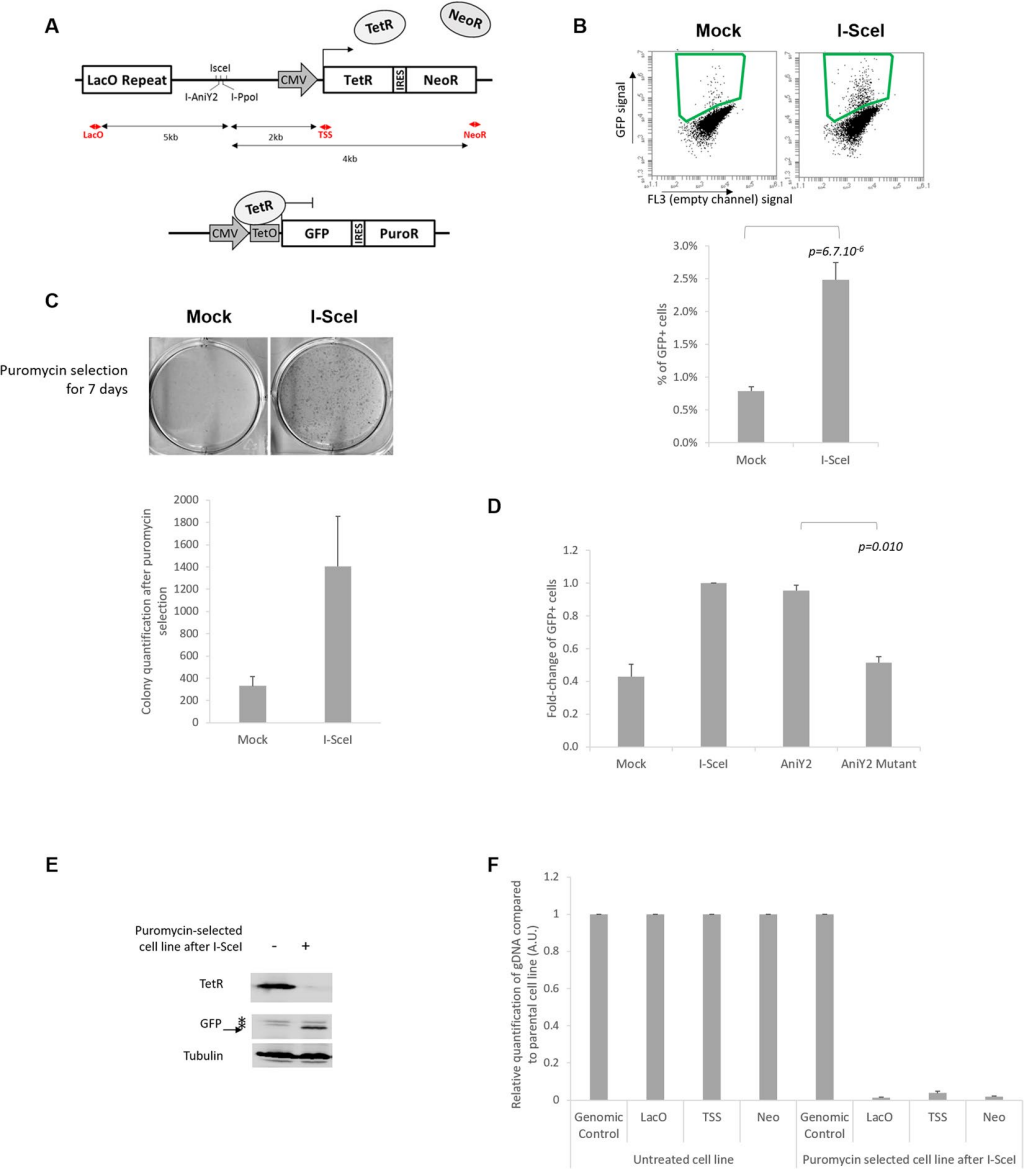
Two-component system to study large deletions following a DSB: (**A**) Schematic representation of the cell line, named U2OS-RE-TetR-GFP. Two components have been stably integrated in the U2OS cell line: the first (top panel) is composed of LacO repeats, an array with specific RE sites for I-SceI, I-PpoI and I-AniY2, and a TetR-IRES-NeoR gene under control of a CMV promotor; the second component (bottom panel) is a bicistronic GFP-IRES-PuroR cassette under the control of two TetO sites. The red arrows indicate the location of the primers used in F, and the black arrows the distance to the RE array. (**B**) Flow cytometry analysis of the GFP-positive subpopulation seven days after I-SceI induction. Top panel: representative dot plots of FACS analysis seven days after I-SceI induction in I-SceI-transfected or in control cells (Mock). The green square indicates the gate used to quantify the percentage of positive cells. Bottom panel: quantification of the percentage of GFP-positive cells seven day after I-SceI induction (n = 11 for a single U2OS-RE-TetR-GFP clone (mc#5). (**C**) Clonogenicity assay: 3 days after I-SceI induction cells were treated with puromycin for one week, then fixed and stained with brilliant blue (top panel). Bottom panel: ImageJ quantification of colony numbers (n = 3). (**D**) The loss of TetR expression is dependent on DSB: fold-change of GFP-positive cells induced by different RE, as indicated (I-SceI, I-AniY2 wt and the nickase mutant I-AniY2-K227M) normalised to I-SceI (n = 3). (**E**) Immunoblot analysis of a polyclonal cell line selected by puromycin treatment following I-SceI induction, with specific antibodies directed against TetR, GFP and tubulin (as a loading control). (**F**) Relative quantification of genomic DNA in 3 independently established puromycin-selected cell lines evaluated by qPCR using specific primers localized around the break site (as indicated in A), compared to a genomic control region (Genomic control #1) and normalized to the signal from untreated cells (n = 3). All *p*-values are from two-tailed, paired T-tests. All error bars represent the standard error of the mean, unless stated otherwise.

To study the long-term and inherited effect of DNA DSB repair on gene expression, we established a two-component system allowing the quantification of long-term loss of gene expression close to DNA damage. The U2OS cell line was created by stable integration of two independent sequences (Fig. 1A and S1A). The first insertion is composed of a restriction endonuclease (RE) site array (containing recognition sites for the rare-cutter enzymes I-SceI, I-PpoI and I-AniY2) localized 2 kb upstream of an actively transcribed bicistronic cassette coding for the TetR and Neomycin-Resistance (NeoR) genes under control of the CMV promotor. The second component is a bicistronic cassette coding for a nuclear GFP and the Puromycin-Resistance (PuroR) genes under the control of a TetO cassette. The TetR protein, expressed by the first component, represses the GFP and the PuroR (Figure S1B). This system is reversible either by doxycycline disruption of the TetR:TetO interaction (Figure S1B) or by loss of the TetR protein.

To induce the site-specific DSB, cells are transiently transfected with a plasmid coding for a nuclear-localisation inducible form of I-SceI (I-SceI-GR-LBD)^24^: the nuclease is re-localized from the cytoplasm to the nucleus upon triamcinolone acetonide (TA) hormone treatment (2 hours) (Figure S1C). Nuclear entry is associated with activation of the local DNA damage response, as indicated by γH2AX and 53BP1 foci adjacent to the lacO array (Figure S1D). To evaluate the percentage of breaks occurring after I-SceI nuclear induction, the genomic DNA was extracted and the RE array amplified by qPCR, alongside a genomic control region. A DSB is associated with a lack of amplification of the RE array. Under our experimental conditions, around 35% of cells contain an unrepaired DSB at the I-SceI sites two hours after DSB induction (Figure S1E). This new two-component system allows the quantification and characterization of long-term loss of gene expression induced by a DSB.

### A double-strand break induces loss of TetR expression

Strikingly, following site-specific DSB and repair, a new population of cells characterized by the expression of the bicistronic cassette GFP-IRES-PuroR appears. This GFP-positive population was quantified by fluorescence-activated cell sorting (FACS) analysis seven days after I-SceI-induced DSB (Fig. 1B), or by a clonogenic survival assay, following puromycin selection (Fig. 1C). It is important to note that this phenomenon appears to be independent of the chromosomal insertion location of the cassette, as this result has been reproduced in six different polyclonal cell lines (independently established) as well as in thirteen different monoclonal cell lines (Figure S1F,G). It is also independent of the LacO repeat sequences (Figure S1H). Furthermore, the appearance of this subpopulation is dependent on RE cutting: it was not observed in a cell line where the RE array was deleted (Figure S1F). We obtained similar results with other site-specific endonucleases: I-PpoI (Figure S2) and I-AniIY2 (Fig. 1D). Interestingly, the expression of the nickase mutant I-AniIY2-K227M which can induce only a single-strand break^25,26^ was not associated with the appearance of this GFP-expressing subpopulation (Fig. 1D).

All together, these data suggest that after DSB and repair, a subset of cells (<10%) lose the expression of the neighbouring gene. This could be due to long-term silencing mediated by a change of the local chromatin state^27^ or simply by a large deletion including the neighbouring gene^28,29^.

### A double-strand break induces large deletions

In order to investigate the mechanism of loss of expression after DSB, puromycin selection was employed to isolate the subpopulation of cells expressing GFP-IRES-PuroR after DNA damage. Antibiotic selection after DSB induction gave polyclonal cell lines characterized by expression of GFP and the absence of the TetR protein (Fig. 1E). Genomic DNA was extracted and sites proximal to the DSB site were compared to a distant control region by quantitative PCR (qPCR) assay (sites annotated in Fig. 1A)^30^. The qPCR signals obtained for each set of primers were normalized to the signal from the parental cell line, i.e. the cell line without DSB induction and puromycin selection. Interestingly, the results from three independently established polyclonal cell lines show a near complete loss of DNA template around the DSB site (Fig. 1F), demonstrating that the loss of TetR is caused predominantly by large deletions of at least 9 kb. To confirm this result, we also isolated clones showing GFP appearance after DSB by limited dilution, without antibiotic selection, into 96 well plates. After plating, GFP-expressing clones (≤1 per well) were selected through puromycin resistance and colonies derived from single cells (Figure S3A) were investigated for deletions. The qPCR assay was carried out as above and a similar pattern of loss of DNA was observed on 43 different GFP-positive clones (Figure S3B,C).

Our data indicate that we have established a new tool to study the mechanism behind large, DSB-dependent, deletions, in contrast to the majority of I-SceI systems which are only designed to monitor deletions up to a certain size, e.g. 500 bp from the I-SceI cut site^31^. Given that we never observed GFP-positive cells without a corresponding deletion, for brevity we refer to I-SceI-dependent increases in GFP-positive cells as ‘I-SceI-dependent deletions’.

### The DSB-induced large deletions are independent of ATM, ATR and DNA-PK activation and cell cycle stage at the time of damage

**Figure 2:**
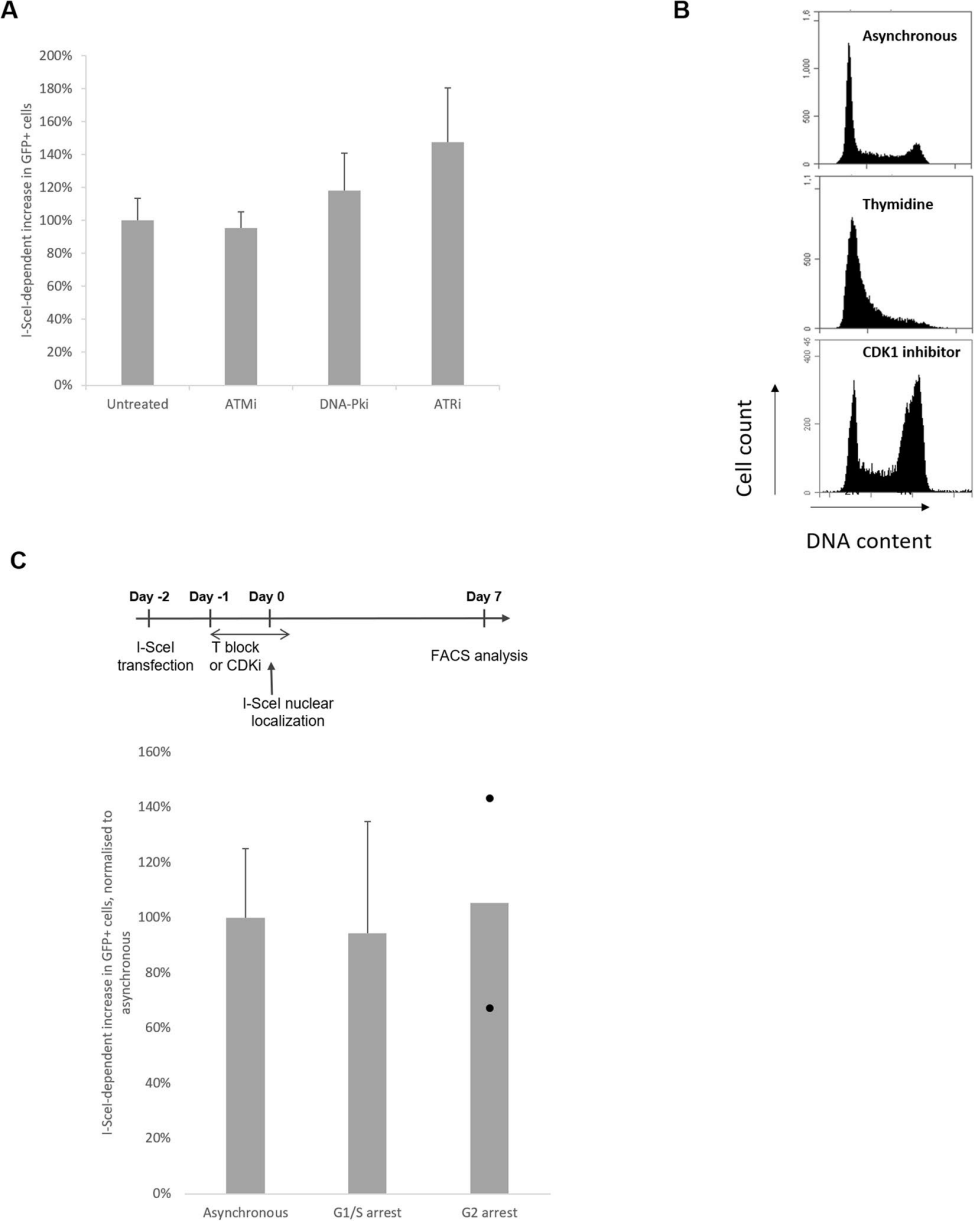
The DSB-induced deletions are independent of ATM, ATR and DNA-PK activation and DNA replication at the time of damage: (**A**) I-SceI-dependent increase in deletions in cells treated with or without inhibitors from 1 h before DSB induction (and until 24 h after for ATM and DNA-PK inhibitors, 4 h after for ATRi), normalised to control cells (n = 3 for ATMi and DNA-PKi, n = 4 for ATRi). (**B**) Cell cycle profile quantifying PI-stained DNA of proliferating cells (top panel), thymidine-arrested cells (G1/S; middle panel) or CDK1-I-treated cells (RO-3306; G2; bottom panel); (**C**) DSB-induced deletions are independent of cell-cycle stage at the time of damage. Top panel: experimental design to study the impact of cell-cycle stage at the time of DSB induction on deletion: after I-SceI transfection, cells are arrested with 18 h treatment with the drug pre-I-SceI induction, as indicated. During the arrest, I-SceI nuclear localisation is induced. Cells are released 4 hours after the induction. Bottom panel: I-SceI-dependent increase in deletions normalised to asynchronous cells (asynchronous n = 5; G1/S arrested (thymidine) n = 3; G2 arrested (CDKi) n = 2, each dot represents one experiment).

We first tested if this deletion requires activation of early damage response kinases. Using inhibitors of the kinases ATM, DNA-PK and ATR (Figure S4A,B) we did not observe significant changes in the levels of large deletions (inhibition from one hour before I-SceI nuclear localization until 24 h after damage induction for ATM and DNA-PK inhibitors or 4 h after for the ATR inhibitor; Fig. 2A). This observation suggests that the activation of these canonical kinases at the time of the DSB is not required for this phenomenon.

Secondly, we hypothesized that collision between DSB repair and DNA replication could be a cause of this genomic instability. To test the role of replication fork progression in the appearance of DSB-induced deletions, the I-SceI cutting was carried out in arrested cells. Cells were arrested either at the G1/S phase boundary by thymidine treatment, or in G2 phase by CDK1 inhibitor treatment (Fig. 2B). TA treatment of arrested cells allowed I-SceI nuclear localisation and, after 4 hours to allow damage and repair, cells were released. After seven days, we did not observe any significant change in the population of I-SceI-dependent GFP-positive cells that were arrested at the time of damage, compared to asynchronous cells (Fig. 2C). This suggests that the deletions are not restricted to cells undergoing DNA replication at the time of damage.

### R-loop modulators alter DSB-induced deletion frequencies

**Figure 3:**
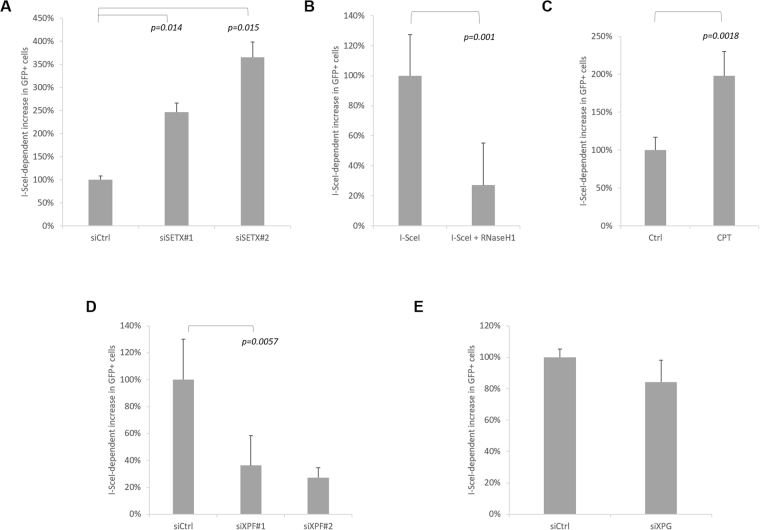
R-loop modulators alter DSB-induced deletion frequencies: (**A**) Knockdown of the helicase Senataxin increases the level of DSB-induced deletions. I-SceI-dependent increase in deletions in Senataxin-depleted cells normalised to siCtrl cells (n = 3). (**B**) RNAseH1 expression prevents DSB-induced deletions: I-SceI-dependent increase in deletions in RNaseH1-expressing cells normalised to control cells (n = 4). (**C**) Camptothecin (CPT) treatment increases DSB-induced deletions: I-SceI-dependent increase in deletions in CPT treated cells normalised to control cells (n = 7). (**D**,**E**) Knockdown of the endonucleases XPF/ERCC4 (**D**) and XPG/ERCC5 (**E**). I-SceI-dependent increase in deletions four days after I-SceI induction in XPF/ERCC4-depleted cells (siXPF) normalised to control cells (siCtrl) (n = 6 for siXPF#1; n = 3 for siXPF#2, siXPG). Knockdown efficiencies shown in Figure S5A.

R-loop structures, associated with transcription, have been identified as an important source of genetic instability^9,10^. We hypothesised that these molecular structures could be one of the causes of our deletions. To test the hypothesis that DNA:RNA hybrid are involved in DSB-dependent large deletions we employed three approaches. We first asked whether knockdown of Senataxin, an DNA:RNA helicase, capable of resolving DNA:RNA hybrid^21^, would alter the level of deletions. After depletion of Senataxin I-SceI-dependent deletions are significantly increased (Fig. 3A, S5A,B). We next over-expressed RNaseH1 (Figure S5C), an enzyme capable of removing transcription-associated DNA:RNA hybrids^11,32,33^. This resulted in a strong (80%) reduction in I-SceI-dependent deletions (Fig. 3B, S5D). To control for possible confounding effects, we confirmed that RNaseH1 over-expression did not reduce the cutting efficiency of I-SceI (Figure S1E), or alter the level of transcription of the TetR-IRES-Neo gene (Figure S5E). Finally, we inhibited TopI to increase negative supercoiling behind the transcription complex, an approach that has previously been shown to increase R-loops^34,35^. TopI inhibition with camptothecin (CPT) induced a two-fold increase in DSB-associated deletions (Fig. 3C, S5C,F). In addition to increasing negative supercoiling, CPT-stabilised TopI-cleavage complexes lead to DSBs upon collision with the DNA replication machinery^36^. To control for a possible CPT damage-dependent effect on I-SceI-dependent deletions, we carried out TopI knockdown; this is expected to increase transcription-generated negative supercoiling in the absence of stabilised TopI-cleavage complex damage. TopI knockdown (Figure S5G) also resulted in an increase in DSB-dependent deletions, similar to that seen with CPT (compare Figure S5F and G).

R-loops are a 3-stranded structure: it has been shown that this structure can be a target for structure-specific endonucleases such as XPF/ERCC4 and XPG/ERCC5^13,37^, as part of the transcription-coupled nucleotide excision repair (TC-NER) pathway. To study the influence of these endonucleases, XPF and XPG were depleted by siRNA in our DSB deletion reporter system. The depletion of XPF led to a significant decrease in deletions, suggesting a role for this endonuclease in the DSB deletion mechanism (Fig. 3D and S5A,H). By contrast, the depletion of XPG by siRNA did not prevent DSB-induced deletion (Fig. 3E and S5A,I). In addition, the depletion of ERCC8, a subunit of CSA^38^, involved in the early stages of TC-NER upstream of XPF/XPG activity, did not affect the DSB-induced deletion (Figure S5J). These data suggest an NER-independent role of XPF, which also has roles in alternative error-prone and deletion associated DSB repair pathways, namely alternative end joining (Alt-NHEJ) and single strand annealing (SSA)^5,39–42^.

Overall, the SETX, RNaseH1 and TopI data are consistent with a role for R-loops in our DSB-dependent deletions. We next asked whether inhibiting transcription reduced the level of DSB-dependent deletions and whether R-loops could be detected locally by DNA:RNA immunoprecipitation (DRIP).

### Deletions are unaffected by modulation of transcription and R-loops are not detected by DRIP in undamaged cells

**Figure 4:**
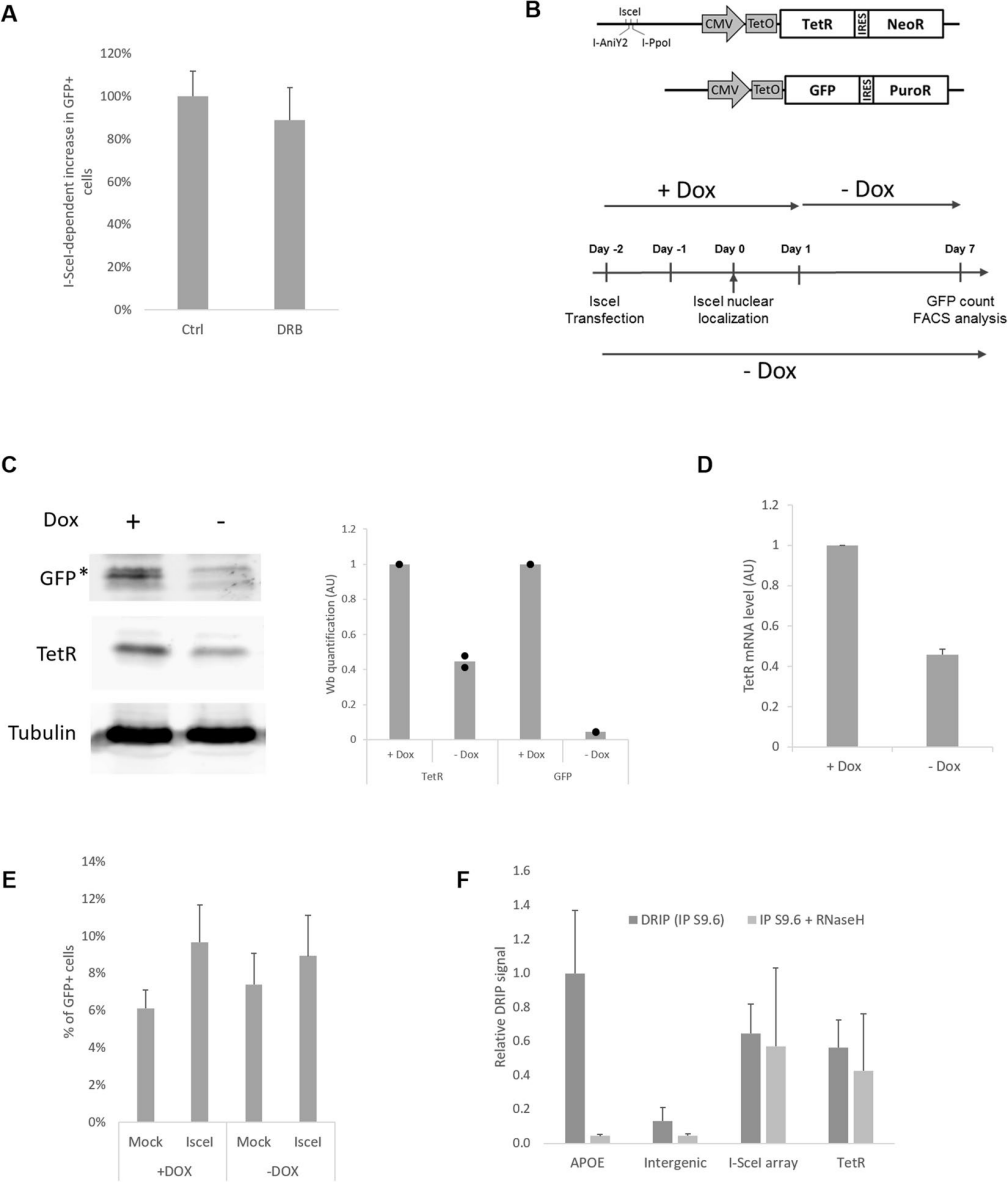
DSB-dependent deletions are unaffected by modulation of transcription: (**A**) I-SceI-dependent increase in deletions seven days after I-SceI induction in cells treated with the transcription inhibitor DRB compared to control cells (n = 7). (**B**) Schematic representation of the transcription regulation system. Upper panel: two TetO cassettes are inserted in front of TetR-IRES-NeoR. Lower panel: experimental design to study deletions in a context of high (+Dox) or low (−Dox) transcriptional activity, indicating I-SceI transfection and induction, doxycycline treatment and FACS analysis. (**C**) Immunoblot analysis of the cell line treated with or without doxycycline for six days, using antibodies against TetR, GFP and tubulin (as a loading control) (left panel). Right panel: relative quantification (n = 2). (**D**) TetR mRNA quantification by RT-qPCR, normalized to GAPDH mRNA level (n = 4). (**E**) Percentage of GFP-positive cells after I-SceI induction in high (+Dox) or low (−Dox) transcriptional activity context (n = 3). (**F**) DRIP-qPCR analysis of DNA:RNA hybrid structure at TetR-IRES-NeoR gene in undamaged cells (no I-SceI induction). Primers targeting the APOE gene are used as a positive control for R loop formation, and primers specific to an intergenic region are used as a negative control. The values, corresponding to the signal following S9.6 IP of isolated DNA (dark grey bar) or of *in vitro* RNaseH-treated DNA (clear grey bar), are represented as fold increase normalised to the APOE positive control (n = 7).

To test the influence of transcriptional activity on our phenotype, cells were first treated with an inhibitor of transcription elongation, DRB, one hour before and concomitant with DSB induction (2 h)^43^. This short and global transcription elongation inhibition did not significantly reduce the level of deletions (Fig. 4A; S6A).

Transient global inhibition of transcription is a crude tool: to more selectively study the potential role of local transcriptional activity on deletion, our two-component system was modified by integration of two TetO cassettes between the CMV promotor and the TetR-IRES-PuroR gene (Fig. 4B). This modification allows regulation of the gene transcription activity: in the presence of doxycycline, the interaction between TetR and the TetO cassette is prevented, and consequently the TetR gene is highly expressed, as is the GFP gene. In contrast, when doxycycline is removed, there is an auto-repression of TetR transcription by the TetR protein (schematic in Figure S6B, GFP expression in both condition Figure S6C); this results in a 60% decrease of TetR protein level (Fig. 4C) and a corresponding drop in mRNA level (Fig. 4D). This system allows deletion quantification with the same cell line, in a context of high (+Dox) or low (−Dox) transcription. It is important to note that for the high expression level condition, the doxycycline was removed 24 h after TA induction. This allows a high level of transcription during the break and the repair, and subsequent repression of GFP over the next six days, prior to scoring GFP-positive cells (Fig. 4B). Induction of DSBs in the context of high or low transcription is equally efficient (Figure S6D) and led to similar levels of deletions (Fig. 4E). This result suggests that the level of expression of the neighbouring gene does not play a major role in misrepair deletions, in concordance with the literature^44^.

To evaluate the presence of DNA:RNA hybrids at the highly expressed TetR-IRES-NeoR gene, immunoprecipitation with the DNA:RNA specific antibody S9.6 followed by qPCR analysis (DRIP-qPCR) was performed (cell line in Fig. 1). While we detect a specific R-loop signal at the APOE positive control locus^12,33^, we did not detect R-loop levels above background at the TetR gene (background estimated by treatment with RNaseH *in vitro*, pre-IP; Fig. 4F). Based on the transcription-level independent nature of the deletions, and the lack of detectable R-loops, we conclude that canonical R-loop processing is not responsible for the DSB-dependent deletions.

### DNA:RNA hybrids occur at the break site

**Figure 5:**
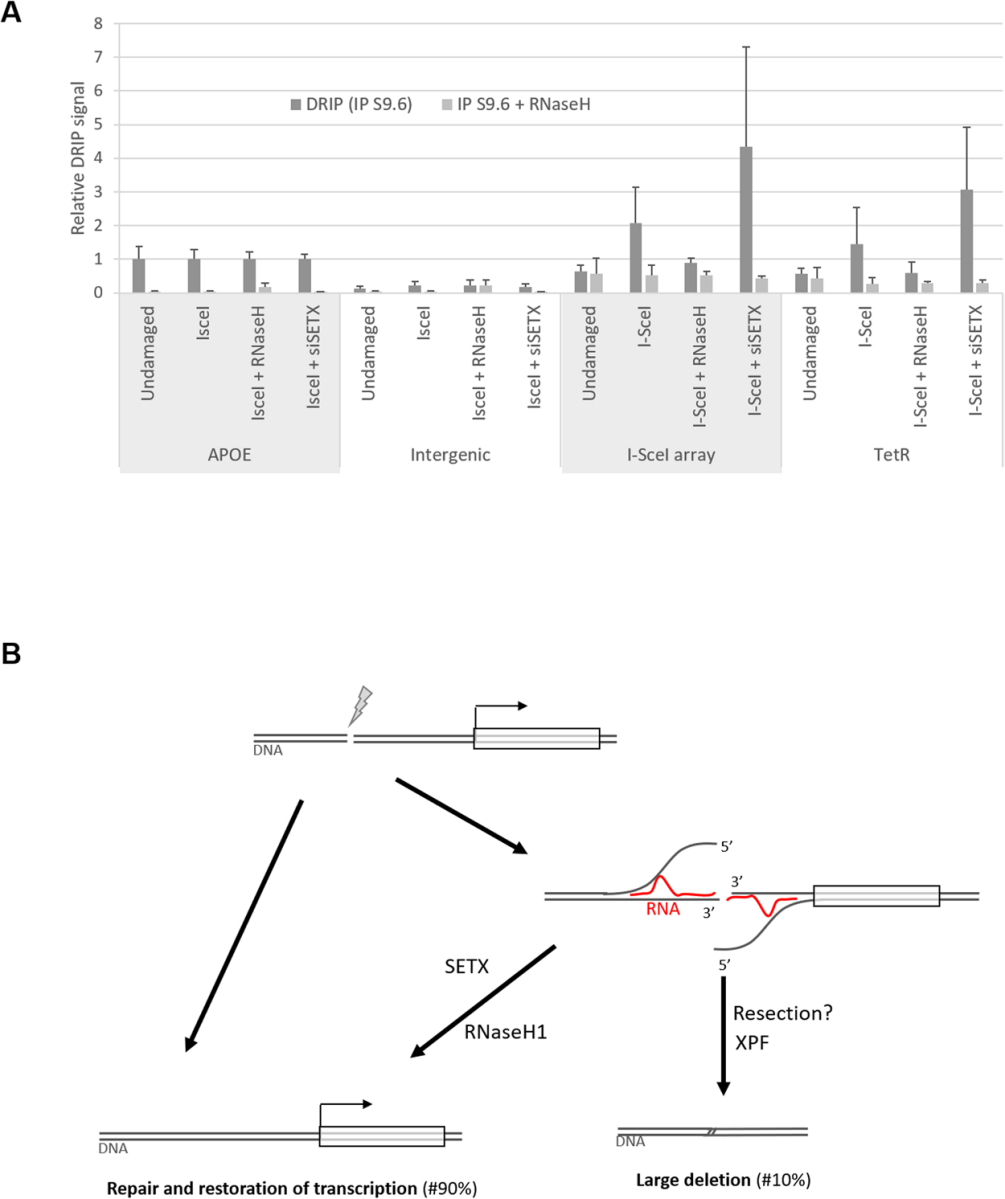
DNA:RNA hybrids occur at the break site: (**A**) DRIP-qPCR analysis of DNA:RNA hybrid structure at TetR-IRES-NeoR gene in undamaged cells or after DSB induction. Primers targeting the APOE gene are used as a positive control for R loop formation, and primers specific to an intergenic region are used as a negative control. The values, corresponding to the signal following S9.6 IP of isolated DNA (dark grey bar) or of *in vitro* RNaseH-treated DNA (clear grey bar), are represented as fold increase normalised to the APOE positive control (Undamaged, n = 7 (from Fig. 4(F)); I-SceI, n = 7; I-SceI + RNaseH1, n = 3; I-SceI + siSETX, n = 4). (**B**) Model of DSB-induced large deletion dependent on a DNA:RNA hybrid associated with transcription from the DSB: Generally a DSB occurring in close proximity to a gene is efficiently repaired, either by HR or NHEJ, and the transcription program is not affected over the long term (left side). Alternatively (right side), transcription and DNA:RNA hybrid generation displaces the 5′ DNA strand. Senataxin is shown reversing this, promoting correct repair. ERCC1/XPF is required to cleave the displaced 3′ DNA strands.

We next considered the possibility that DNA:RNA hybrids were generated as a consequence of local transcription occurring after the I-SceI DSB. Two hours after I-SceI induction, cells were collected and DRIP was performed. We detected an increase in DNA:RNA signal after I-SceI cleavage adjacent to the I-SceI array and at the TetR gene but not at the intergenic control region or APOE gene (Fig. 5A). The DSB-dependent DRIP signal was increased further after Senataxin knockdown (Fig. 5A, IsceI + siSETX condition). By contrast, DNA:RNA hybrids at the break site were prevented by RNaseH1 over-expression (Fig. 5A, IsceI + RNaseH1 condition). These trends, while not statistically significant, due to large variability in the DRIP signal, are consistent with a role for DSB-dependent DNA:RNA hybrids in the deletion process.

## Discussion

In this report, we have established a cell line to study DSB-induced large deletions based on the loss of a gene in the proximity of an RE array. The fact that TetR sequence was missing in all the clones analysed (Figure S3) strongly suggests that silencing due to epigenetic changes, as previously described for a system utilising a promoter known to undergo DNA methylation-dependent silencing^27^, does not account for the loss of TetR expression in our system. Large deletions induced by I-SceI have previously been observed^28,29^. However the requirement for selection to observe these large deletions usually precludes an estimate of frequency and the mechanism resulting in these large deletions has not been determined^28,29^. The gain of GFP-expression associated with deletions in our system allows the quantification of rare events. We observe the frequency of DSB-induced large deletions to vary between 0.3% and 22% of transfected cells, across nineteen independently integrated cell lines (Figure S1F; taking into account background, I-SceI independent loss and transfection efficiency of ~35% (Figure S1D). These frequencies are not per DSB: I-SceI will cut repeatedly, until a misrepair event removes the cleavage motif, therefore the rate of deletion per DSB will be lower than the rate per transfected cell.

LacO repeats, in the presence of LacI repressor, have been shown to act as fragile sites, generating DSBs^45^. In our system we are confident that the lacO repeats are not playing a significant role in the DSB-induced deletion for three reasons: (i) our experiments are carried out in the absence of LacI protein (with the exception of the co-localization experiment with γH2AX and 53BP1 in S1C); (ii) we inserted only 59 LacO repeats, fewer than the 256 repeats shown to generate a fragile site^45^; (iii) the DSB-induced large deletions are independent of replication fork progression at the time of I-SceI cleavage (Fig. 2C). Furthermore, we generated a cell line without LacO repeats and found the frequency of DSB-induced deletions to be unchanged compared to the original cell line, as expected (Figure S1H).

The detection of DNA:RNA hybrid formation at a DSB is in agreement with a recent observation in fission yeast^14^, and laser-stripe damage-dependent accumulation of DNA:RNA hybrid in mammalian cells^15^. DNA:RNA hybrids formation at the break site could be explained by the previously documented local initiation of transcription in response to the DSB^16–18^. The lack of effect of transient DRB treatment on the observed deletions may indicate a delayed or non-canonical transcriptional activity at the DSB: DRB acts by inhibiting the CDK9-dependent transition of PolII from initiation to elongation^46^. Ohle *et al*. found that efficient removal of RNA:DNA hybrids was required for homologous recombination repair and viability after DSB induction in fission yeast^14^. This shared link between RNA:DNA hybrid removal and DSB repair is intriguing, however while controlled levels of RNA:DNA hybrids in the *S. pombe* system appear to promote repair, it is unclear what physiological role RNA:DNA hybrids play in the repair of our I-SceI DSB.

A DSB flanked by homologous sequences may undergo single-strand annealing (SSA), including XPF cleavage, generating a deletion^5,47^. The speculative model we propose involves mis-repair of a targeted DSB associated with DNA:RNA hybrid processing. Transcription from the DSB end will displace the 5′ end of the DNA, promoting SSA. SSA entails resection of the 5′ end until homologous sequences are revealed. This is followed by annealing and subsequent cleavage of 3′ overhangs by XPF to complete the deletion (Fig. 5B). Alternatively, Senataxin can reverse the DNA:RNA hybrid at an early stage, before resection occurs. While the model involving SSA has the advantage of linking XPF activity with a DSB-linked deletion, we have no evidence that SSA occurs in our system, and other models are possible. For instance, it is possible that the subset of breaks generating a large deletion are repaired slowly^48,49^, and loss of Senataxin may be destabilising DNA replication forks^50^, promoting deletions at these sites of repair. Genetic instability is a common feature of most types of cancer^51^. Deletions of between 1–100 kb are a signature of BRCA1 and 2-negative breast cancers^52^. Deletions may in most cases be tolerable to a cell, as indicated by the surprisingly high proportion of post-mitotic neurons containing Mb-scale deletions, revealed by single-cell sequencing^53^. Nonetheless, loss of DNA repair or tumour-suppressor genes will contribute to the development of further genetic instability or cancer.

Altogether, data from our two-component system suggests DSB-induced DNA:RNA hybrid formation may be mechanistically associated with a minor mis-repair pathway generating large deletions.

## Experimental Procedures

### Plasmids

The plasmid pcDNA4-GFP-IRES-PuroR was generated by insertion of the bicistronic cassette GFP-IRES-PuroR (amplified from pGIPZ-GFP(nls)-IRES-PURO (Murray lab, University of Sussex)) in pcDNA4-CMV-TetO (Invitrogen).

The plasmid pIRES-LacOR-REsites-TetR-IRES-NeoR was generated in three steps: (i) TetR gene (amplified from pcDNA6 (Invitrogen) was inserted in pIRESneo3 (Clontech) (ii) The LacO repeats were integrated: 16 LacO repeats (amplified from the plasmid Holo16 (Sweet lab, University of Sussex)) and 43 LacO repeats (from PLAU43^54^) were inserted in pIRESneo3-TetR (iii) The RE sites array containing specific sequence for I-SceI (3 times), AniI-Y2 and Ppo-I (synthesized by Invitrogen) was integrated in the plasmid obtained at step (ii).

The pIRES-LacOR-REsites-TetO-TetR-IRES-Neo was generated by integration of two TetO cassettes (generated by Thermofisher) in pIRES-LacOR-REsites-TetR-IRES-Neo.

All primer sequences and cloning details are available on request.

Other plasmids used in this study are pdsRED-I-SceI^24^, pCVL-HA.NLS.I-AniIY2wt, pCVL-HA.NLS.I-AniIY2-K227M^25^, pBABE-IPpoI (Puromycin resistant gene was removed.), pCMV6-AC-RNaseH1 (O. Wells, University of Sussex), pLacI-GFP (Savic Lab, University of Sussex).

### Cell culture, DNA transfection, establishment of stable cell lines, siRNA transfection and drug treatment

U2OS cells were obtained from ATCC, tested for mycoplasma contamination and grown in Dulbecco’s modified Eagle’s medium (Gibco) supplemented with 10% foetal bovine serum (PAN biotech), Penicillin/Streptomycin (Corning) and L-Glutamin (Gibco). All plasmid transfections utilised the Jet-Pei transfection reagent (Polyethylenimine 25000, PolyScience) as previously described^30^.

The “*U2OS RE-Sites TetR GFP”* cell line was generated in two steps: (i) stably integration of pcDNA4-GFP-IRES-Puromycin through transfection followed by a Puromycin selection (Sigma, 2.5 µg/ml) then (ii) stably integration of HpaI-linearized pIRES-LacOR-REsites-TetR-IRES-Neo through G418 selection (200 μg/ml). Monoclonal cell lines were generated by limited dilution.

Cells expressing PpoI were established after transduction with retroviral vectors. Virus production and cell infection were performed as previously described^30^.

Smart-pool siRNA (siCtrl, siSETX#1, siTOP1, siXPF#1, siERCC8) and individual siRNA (siSETX#2, siXFP#2, siXPG) were ordered from Dharmacon (see Supp. Table 1: references and sequences). All siRNA transfections were done with Lipofectamine RNAimax (Invitrogen) following the manufacturer’s instructions.

The chemical compounds (and their final concentrations) used in this study were: ATMi: Ku-55933 (Abcam; 10 μM), ATRi: VE-822 (STRATECH SCIENTIFIC; 10 µM), Camptothecin (Sigma; 5 μM), CDK1i: RO-3306 (Sigma; 10 µM) DNA-PKi: NU-7026 (Abcam; 20 μM), Doxycycline cyclate (Sigma; 2 μg/ml), 4OH-Tamoxifene (Sigma; 25 μM), Triamcinolone acetonide, TA (Sigma; 1 μM), and thymidine (Sigma; 2.5 mM).

### Fluorescence-activated cell sorting (FACS) analysis

For quantification of GFP-positive cell populations, cells were trypsinized and re-suspended in complete media. Samples were run on a FACS-accuri (Beckton Dickinson) and data analysed with the BD accuri software. Briefly single cells were gated, first on their size (FLH) and their granularity (SSC) to exclude debris, and then on the linearity between FLH-H and FLH-A signal to exclude doublets. GFP-positive cells were quantified on the signal read on FL1 detector (GFP) vs FL3 (empty channel).

For cell-cycle analysis, cells were fixed with cold ethanol 70%, washed with PBS and re-suspended in PBS containing propidium iodide (PI, Sigma, 5 ug/ml) and RNAse A (Sigma, 50 ug/ml) overnight at 4 °C. Samples were run and single cell gated as described above. PI signal (correlating with DNA content) was read on FL2 detector.

### DSB-dependent deletion reporter system

The “*U2OS RE-Sites TetR GFP”* cell lines were seeded at 70% confluency and transfected with I-SceI-GR-LBD plasmid, as described above. DMEM media phenol-free (Gibco) with charcoal-stripped serum (Gibco) was used to prevent premature nuclear-localisation of I-SceI-GR-LBD. Two days after transfection, cells were treated with the drug triamcinolone acetonide (TA) (Sigma, 1 μM) for 2 to 4 hours. After TA induction, cell were kept in culture, collected at different days (Day 4 and 7), and GFP-positive cells quantified by FACS, as described above. For the analysis of the GFP-positive subpopulation, data can be represented either by the raw percentage of GFP-positive cells, or by the fold increase of the GFP subpopulation normalised to undamaged cells $(Fold\,increase=\frac{[ \% \,of\,GFP\,sample\,]}{[average\, \% \,of\,GFP\,untreated\,condition]})$, or by percentage of GFP-positive cells with subtraction of background levels (mock), normalised to a reference condition, $({\rm{I}}-{\rm{SceI}}-{\rm{dependent}}\,{\rm{increase}}\,{\rm{in}}\,{\rm{GFP}}+{\rm{cells}}=\frac{([ \% \,{\rm{of}}\,{\rm{GFP}}\,{\rm{I}}-{\rm{SceI}}\,{\rm{sample}}]\,-[ \% \,{\rm{of}}\,{\rm{GFPMock}}\,{\rm{sample}}]\,)}{({\rm{average}}\,{\rm{of}}\,[[ \% \,{\rm{of}}\,{\rm{GFP}}\,{\rm{I}}-{\rm{SceI}}\,{\rm{reference}}]-[ \% \,{\rm{of}}\,{\rm{GFPMock}}\,{\rm{reference}}]])}\ast 100).$

siRNA depletion in DSB-induced deletion reporter system: cells were first transfected with siRNA overnight (as described above), the day after, cells were washed and transfected with I-SceI-GR-LBD, 48 h later TA induction was as described above. The quantification of a GFP-positive subpopulation was as described above.

Kinase inhibition (ATM, ATR, DNA-PK): 48 h after I-SceI transfection, cells were pre-treated for 1 h with the chemical inhibitor (as described above), then I-SceI nuclear localisation was induced (as described above) in the presence of the inhibitor and the inhibitor was maintained for 24 h after I-SceI nuclear localisation induction (TA). The GFP-positive subpopulation was analysed as described above.

CPT: 48 h after I-SceI transfection, cells were pre-treated for 1 h with the drug (as described above), then I-SceI nuclear-localisation was induced (as described above) in the presence of the inhibitor. After induction of I-SceI nuclear localisation, cells were washed and inhibitor removed. The GFP-positive subpopulation was analysed as described above.

Replication assay: 24 h after I-SceI transfection, thymidine was added (2.5 mM) for 18 h, and I-SceI nuclear localisation was induced (as described above) in the presence of thymidine. Then cells were washed 3 time with PBS and released. The GFP-positive subpopulation was analysed as described above. Cell cycle arrest and release were monitored by FACS as described above.

### Immunoblot analysis

Proteins were resolved by Mini Gel SDS-PAGE (Bio-Rad system) and transferred to nitrocellulose membrane (GE Healthcare) as previously described^30^. All the blocking and antibody incubations were done in TBS −0.2% Tween-20 5% BSA (Fisher). The following primary antibodies were used: anti-53BP1 (1:1000, Millipore), anti-ATM (1:1000, Abcam), anti-Chk1-phS317 (1:1000, Cell Signalling Technology), anti-GFP (1:1000), anti-HA (1:1000, Sigma), anti-H2AX-P (1:1000, Abcam), anti-p53 (1:1000, DO-1, SantaCruz), anti-p53-phS15 (1:1000, NEB), anti-RNaseH1 (1:1000, Abcam), anti-TetR (1:1000, TETO2, MoBiTec), anti-tubulin (1:5000, Abcam), and appropriate HRP-conjugated secondary antibodies were used: anti-mouse (1:10000, Cell Signalling Technology), anti-rabbit (1:10000, Cell Signalling Technology) and anti-rat (1:10000, Abcam). Immuno-reactive bands were detected by chemoluminescence induced by Supersignal reagent and detected with the ImageQuant LAS 4000 machine (GE Healthcare). Quantification was performed using ImageJ.

### DNA extraction, RNA extraction, qPCR and RT qPCR

Total genomic DNA was isolated using the DNeasy kit (Qiagen). Total RNA was extracted using the RNeasy kit (Qiagen). Reverse transcription was performed by using the Super Script III reverse transcriptase (Invitrogen) and random hexamers (Invitrogen).

The list of primers used for qPCR are available in Supp. Table 2. Quantitative PCR was performed with goTaq qPCR master mix (Promega) and Mx3005-P qPCR machine (Stratagene). The data was analysed with MX-Pro software (Stratagene).

### Immunofluorescence microscopy

Images of GFP-positive live cells were acquired with the AMG-Evos inverted microscope. Immunofluorescence microscopy was performed as described^30^, with antibody dilutions: HA (1/500, Sigma), γH2AX (1/500, Millipore), GFP (1/500, Roche). Samples were examined either with a microscope (Zeiss) equipped with a 10X, a 40X dry objective and a 100X oil immersion objective and a Hamamatsu Orca ER camera, or a confocal microscope (Olympus IX71) equipped with a 40X, 60X and 100X oil immersion objective and a CoolSNAP HQ2 camera. Pictures were analysed with ImageJ software.

### Clonogenicity assay

Two days after I-SceI induction, cells were counted and plated in 6 well plates (200 000 per well). One day after plating GFP+/PuroR clones were selected through puromycin treatment (2.5 µg/ml) for one week. Then the cells were fixed with formaldehyde 3% (FISHER) and stained with Brilliant blue 0.5% (Sigma) in PBS overnight. After PBS washing, drying and scanning, the clones were counted by ImageJ.

### DNA:RNA hybrid Immunoprecipitation (DRIP-qPCR)

After treatment as indicated, cells were collected, and lysed with the lysis buffer (200 mM NaCl, 10 mM Tris pH7.5, 2 mM EDTA, 0.2% SDS and proteinase K 20 µg/ml (Sigma P2308) at 56 °C for 3 h. Then, DNA and associated RNA are precipitated by addition of one volume of isopropanol, washed with ethanol 70%, and resuspended in TE buffer (Tris-HCl pH 7.5, 0.5 mM EDTA). After sonication to obtain DNA fragments less than 800 bp, 50 µg of DNA was treated with recombinant RNaseH (NEB) and used as a negative control. 50 µg of digested DNA was immuno-precipitated with 3 µg of S9.6 antibody^12^ (Kerafast) coupled to IgG magnetic beads (Invitrogen). Washing utilised five buffers (W1: Tris pH8 10 mM, KCl 150 mM, NP40, 0.5%, EDTA 1 mM; W2: Tris pH8 10 mM, NaCl 100 mM, NaDoc 0.1%, TritonX100 0.5%; W3: Tris pH8 10 mM, NaCl 400 mM, NaDoc 0.1%, TritonX100 0.5%; W3b: Tris pH8 10 mM, NaCl 500 mM, NaDoc 0.1%, TritonX100 0.5%; W4: Tris pH8 10 mM, LiCl 250 mM, NaDoc 0.5%, NP40 0.5%, EDTA 1 mM; W5: Tris pH8 10 mM, EDTA 1 mM). After washing, DNA:RNA hybrid associated structures are eluted with SDS buffer and the DNA purified with a Nucleospin Extract II kit (MACHEREY NAGEL). qPCR analyses of DRIP DNAs were performed as described above. The amount of DNA in DRIP samples was extrapolated from analysis of DNA before immunoprecipitation (input) and values were represented as fold increase compared to the positive control.

### DNA break efficiency assay

The U2OS I-SceI TetR GFP cell line was transfected with I-SceI GR-LBD plasmid and its nuclear localisation was induced as described above. Two hours after induction, total genomic DNA was isolated, as described above. For quantification of I-SceI induced cutting efficiency, qPCR was performed (as described above), with amplification across the I-SceI sites. The data was normalised to an unconnected genomic control locus (Genomic control #2), and then expressed as a ratio relative to the undamaged sample. Primer sequences available in Supp. Table 2.

### Statistics

All *p*-values are from two-tailed, paired T-tests. All error bars represent the standard error of the mean, unless stated otherwise.

### Data availability

All datasets generated during and/or analysed during the current study are available from the corresponding author on reasonable request.

## Electronic supplementary material

Supplementary Figures

## References

[1] Mehta, A. & Haber, J. E. Sources of DNA Double-Strand Breaks and Models of Recombinational DNA Repair. *Cold Spring Harbor Perspectives in Biology* **6** (2014). 10.1101/cshperspect.a016428 PMC4142968 25104768

[2] Chang HHY, Pannunzio NR, Adachi N, Lieber MR (2017). Non-homologous DNA end joining and alternative pathways to double-strand break repair. Nat Rev Mol Cell Biol.

[3] Shanbhag NM, Rafalska-Metcalf IU, Balane-Bolivar C, Janicki SM, Greenberg RA (2010). ATM-Dependent Chromatin Changes Silence Transcription In cis to DNA Double-Strand Breaks. Cell.

[4] Sancar A, Lindsey-Boltz LA, Ünsal-Kaçmaz K, Linn S (2004). Molecular Mechanisms of Mammalian DNA Repair and the DNA Damage Checkpoints. Anne. Rev. Biochem..

[5] Bhargava R, Onyango DO, Stark JM (2016). Regulation of Single-Strand Annealing and its Role in Genome Maintenance. Trends in Genetics.

[6] Plessis A, Perrin A, Haber JE, Dujon B (1992). Site-specific recombination determined by I-SceI, a mitochondrial group I intron-encoded endonuclease expressed in the yeast nucleus. Genetics.

[7] Liang F, Han M, Romanienko PJ, Jasin M (1998). Homology-directed repair is a major double-strand break repair pathway in mammalian cells. Proceedings of the National Academy of Sciences.

[8] Moynahan ME, Jasin M (1997). Loss of heterozygosity induced by a chromosomal double-strand break. Proceedings of the National Academy of Sciences.

[9] Sollier J, Cimprich KA (2015). Breaking bad: R-loops and genome integrity. Trends in Cell Biology.

[10] Aguilera A, García-Muse T (2012). R Loops: From Transcription Byproducts to Threats to Genome Stability. Molecular Cell.

[11] Schwab RA (2015). The Fanconi Anemia Pathway Maintains Genome Stability by Coordinating Replication and Transcription. Molecular Cell.

[12] García-Rubio ML (2015). The Fanconi Anemia Pathway Protects Genome Integrity from R-loops. PLoS Genet.

[13] Sollier J (2014). Transcription-Coupled Nucleotide Excision Repair Factors Promote R-Loop-Induced Genome Instability. Molecular Cell.

[14] Ohle C (2016). Transient RNA-DNA Hybrids Are Required for Efficient Double-Strand Break Repair. Cell.

[15] Britton S (2014). DNA damage triggers SAF-A and RNA biogenesis factors exclusion from chromatin coupled to R-loops removal. Nucleic Acids Research.

[16] Francia S (2012). Site-specific DICER and DROSHA RNA products control the DNA-damage response. Nature.

[17] Francia S, Cabrini M, Matti V, Oldani A, d’Adda di Fagagna F (2016). DICER, DROSHA and DNA damage response RNAs are necessary for the secondary recruitment of DNA damage response factors. Journal of Cell Science.

[18] Wei W (2012). A Role for Small RNAs in DNA Double-Strand Break Repair. Cell.

[19] Michalik KM, Böttcher R, Förstemann K (2012). A small RNA response at DNA ends in Drosophila. Nucleic Acids Research.

[20] Liu LF, Wang JC (1987). Supercoiling of the DNA template during transcription. Proceedings of the National Academy of Sciences.

[21] Skourti-Stathaki K, Proudfoot, Nicholas J, Gromak N (2011). Human Senataxin Resolves RNA/DNA Hybrids Formed at Transcriptional Pause Sites to Promote Xrn2-Dependent Termination. Molecular Cell.

[22] Mischo HE (2011). Yeast Sen1 Helicase Protects the Genome from Transcription-Associated Instability. Molecular Cell.

[23] Hatchi E (2015). BRCA1 Recruitment to Transcriptional Pause Sites Is Required for R-Loop-Driven DNA Damage Repair. Molecular Cell.

[24] Soutoglou E (2007). Positional stability of single double-strand breaks in mammalian cells. Nature Cell Biology.

[25] Certo MT (2011). Tracking genome engineering outcome at individual DNA breakpoints. Nat Meth.

[26] McConnell Smith A (2009). Generation of a nicking enzyme that stimulates site-specific gene conversion from the I-AniI LAGLIDADG homing endonuclease. Proceedings of the National Academy of Sciences.

[27] O’Hagan HM, Mohammad HP, Baylin SB (2008). Double Strand Breaks Can Initiate Gene Silencing and SIRT1-Dependent Onset of DNA Methylation in an Exogenous Promoter CpG Island. PLoS Genet.

[28] Honma M (2003). Deletion, rearrangement, and gene conversion; genetic consequences of chromosomal double-strand breaks in human cells. Environmental and Molecular Mutagenesis.

[29] Varga T, Aplan PD (2005). Chromosomal aberrations induced by double strand DNA breaks. DNA Repair.

[30] Tardat M (2010). The histone H4 Lys 20 methyltransferase PR-Set7 regulates replication origins in mammalian cells. Nat Cell Biol.

[31] Soong C-P (2015). Development of a novel method to create double-strand break repair fingerprints using next-generation sequencing. DNA Repair.

[32] Chon H (2013). RNase H2 roles in genome integrity revealed by unlinking its activities. Nucleic Acids Research.

[33] Bhatia V (2014). BRCA2 prevents R-loop accumulation and associates with TREX-2 mRNA export factor PCID2. Nature.

[34] El Hage A, French SL, Beyer AL, Tollervey D (2010). Loss of Topoisomerase I leads to R-loop-mediated transcriptional blocks during ribosomal RNA synthesis. Genes & Development.

[35] Sordet O (2009). Ataxia telangiectasia mutated activation by transcription‐ and topoisomerase I‐induced DNA double‐strand breaks. EMBO reports.

[36] Ryan AJ, Squires S, Strutt HL, Johnson RT (1991). Camptothecin cytotoxicity in mammalian cells is associated with the induction of persistent double strand breaks in replicating DNA. Nucleic Acids Research.

[37] Tian M, Alt FW (2000). Transcription-induced Cleavage of Immunoglobulin Switch Regions by Nucleotide Excision Repair Nucleases *in Vitro*. Journal of Biological Chemistry.

[38] Henning, K. A. *et al*. TheCockayne syndrome group A gene encodes a WD repeat protein that interacts with CSB protein and a subunit of RNA polymerase II TFIIH. *Cell* **82**, 555–564. 10.1016/0092-8674(95)90028-4 7664335

[39] Ciccia A, McDonald N, West SC (2008). Structural and Functional Relationships of the XPF/MUS81 Family of Proteins. Anne. Rev. Biochem..

[40] Bennardo N, Cheng A, Huang N, Stark JM (2008). Alternative-NHEJ Is a Mechanistically Distinct Pathway of Mammalian Chromosome Break Repair. PLoS Genet.

[41] Ma J-L, Kim EM, Haber JE, Lee SE (2003). Yeast Mre11 and Rad1 Proteins Define a Ku-Independent Mechanism To Repair Double-Strand Breaks Lacking Overlapping End Sequences. Molecular and Cellular Biology.

[42] Ahmad A (2008). ERCC1-XPF Endonuclease Facilitates DNA Double-Strand Break Repair. Molecular and Cellular Biology.

[43] Yankulov K, Yamashita K, Roy R, Egly J-M, Bentley DL (1995). The Transcriptional Elongation Inhibitor 5,6-Dichloro-1-β-D-ribofuranosylbenzimidazole Inhibits Transcription Factor IIH-associated Protein Kinase. Journal of Biological Chemistry.

[44] Allen C, Miller CA, Nickoloff JA (2003). The mutagenic potential of a single DNA double-strand break in a mammalian chromosome is not influenced by transcription. DNA Repair.

[45] Jacome A, Fernandez‐Capetillo O (2011). Lac operator repeats generate a traceable fragile site in mammalian cells. EMBO reports.

[46] Wang S, Fischer PM (2008). Cyclin-dependent kinase 9: a key transcriptional regulator and potential drug target in oncology, virology and cardiology. Trends in Pharmacological Sciences.

[47] Al-Minawi AZ, Saleh-Gohari N, Helleday T (2008). The ERCC1/XPF endonuclease is required for efficient single-strand annealing and gene conversion in mammalian cells. Nucleic Acids Research.

[48] Goodarzi AA (2008). ATM Signaling Facilitates Repair of DNA Double-Strand Breaks Associated with Heterochromatin. Molecular Cell.

[49] Löbrich, M. & Jeggo, P. A Process of Resection-Dependent Nonhomologous End Joining Involving the Goddess Artemis. *Trends in Biochemical Sciences* **42**, 690–701. 10.1016/j.tibs.2017.06.011 PMC5604544 28739276

[50] Alzu A (2012). Senataxin Associates with Replication Forks to Protect Fork Integrity across RNA-Polymerase-II-Transcribed Genes. Cell.

[51] Hanahan D, Weinberg RA (2011). Hallmarks of Cancer: The Next Generation. Cell.

[52] Nik-Zainal, S. *et al*. Landscape of somatic mutations in 560 breast cancer whole-genome sequences. *Nature* (2016). 10.1038/nature17676 PMC4910866 27135926

[53] McConnell MJ (2013). Mosaic Copy Number Variation in Human Neurons. Science.

[54] Lau IF (2003). Spatial and temporal organization of replicating Escherichia coli chromosomes. Molecular Microbiology.

