# Supplementary material for: Large XPF-dependent deletions following misrepair of a DNA double strand break are prevented by the RNA:DNA helicase Senataxin
Source: Sci Rep. 2018 Mar 1;8:3850. doi: 10.1038/s41598-018-21806-y (PMC5832799; doi:10.1038/s41598-018-21806-y)
Supplement: Supplementary file 1 — Supplementary Figures [file 41598_2018_21806_MOESM1_ESM.pdf]

Supplementary information

Large XPF-dependent deletions following misrepair of a DNA double strand break are prevented by the RNA:DNA helicase Senataxin

Julien Brustel, Zuzanna Kozik, Natalia Gromak, Velibor Savic and Steve M.M. Sweet

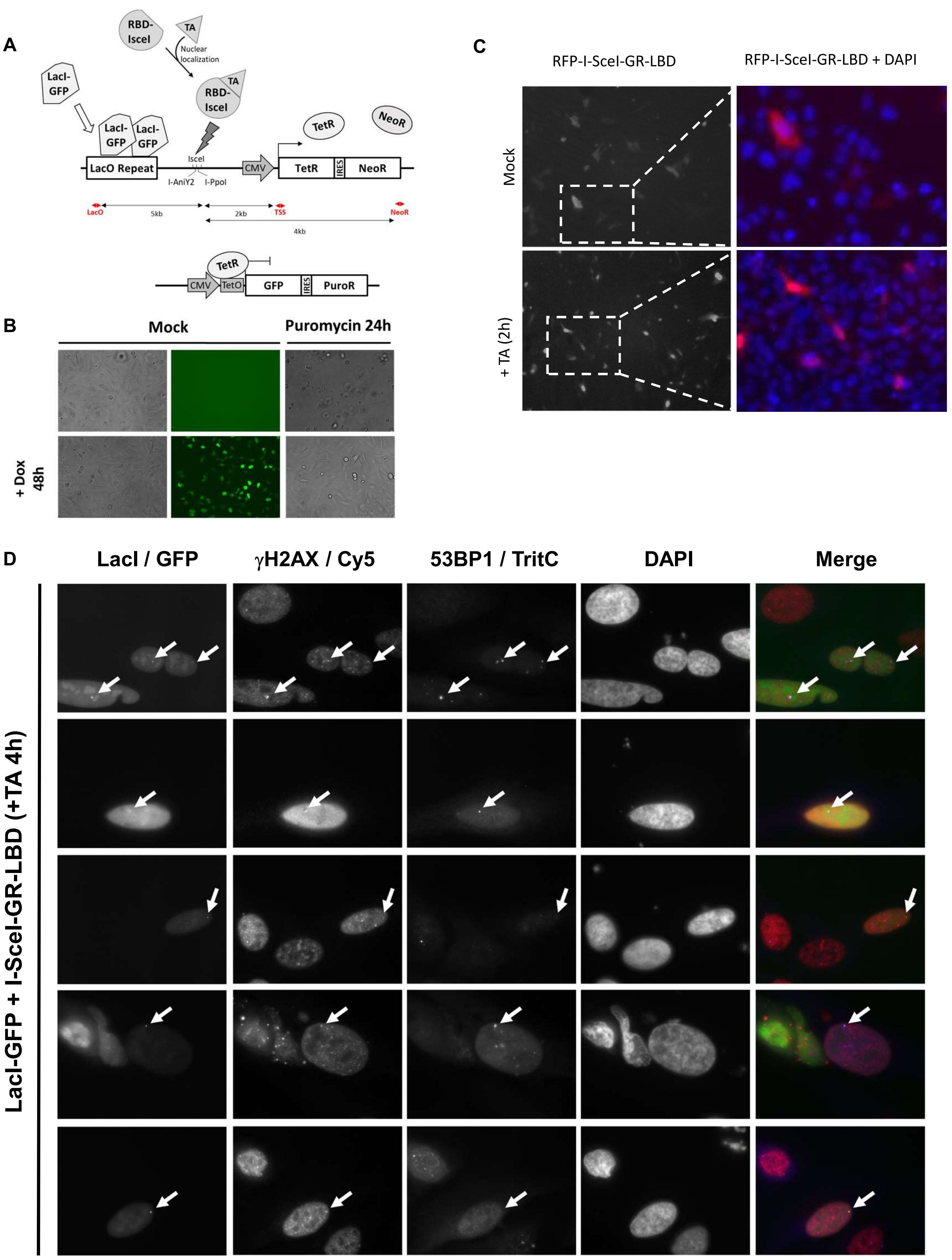

Figure S1. Characterization of the two component system

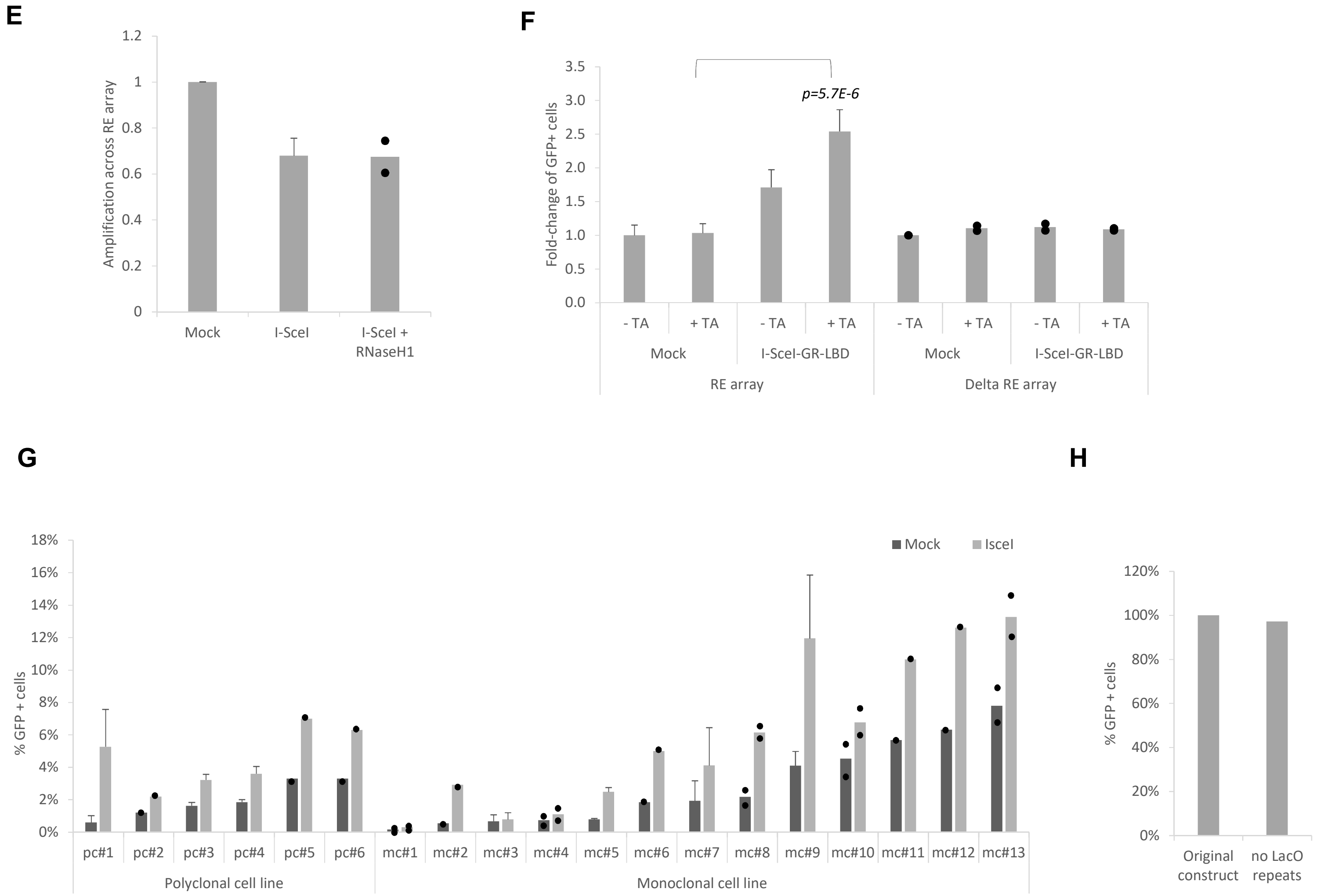

Figure S1: Characterization of the two component system:

(A) Schematic representation of the cell line, named U2OS-RE-TetR-GFP (B) Reversibility of the GFP-IRES-PuroR cassette repression by addition of doxycycline: phase-contrast and fluorescence pictures of U2OS-RE-TetR-GFP cells cultivated with (lower panels) or without (top panels) doxycycline for two days and with (right panel) or without (two left panels) puromycin for 24 hours. (C) Fluorescence images of RFP-tagged I-SceI-GR-LBD two days after transfection with (lower panels) or without (upper panels) nuclear localization mediated by TA treatment. On the left panels: RFP signal, on the right panels: zoom picture corresponding to the white square, RFP signal in red and DAPI signal in blue (D) Immunofluorescence images of U2OS-RE-TetR-GFP cells co-transfected with I-SceI-GR-LBD and LacI-GFP, with TA-mediated nuclear localization of I-SceI; antibodies against GFP, to detect LacI-GFP protein targeted to the LacO repeat localized proximal to the RE array, and γH2AX and 53BP1, to detect DSBs, were employed. Nuclei were stained with DAPI. The white arrows indicate the LacI-GFP foci and the co-localization with γH2AX and 53BP1. (E) Evaluation of cutting efficiency by I-SceI in normal or RNaseH1 expressing cells: 2 hours after I-SceI nuclear induction, cells are collected and DNA extracted. The percentage of cutting is determined by amplification across the RE array by qPCR and normalization to a genomic control region (Genomic control #1) and to undamaged cells (mock). n=3 for I-SceI only; n=2 for I-SceI + RnaseH1 (each dot represents one experiment). (F) GFP-positive population seven days after DSB induction normalised to untreated, in U2OS-RE-TetR-GFP (n=34 experiments on polyclonal and monoclonal cell lines- results for individual cell lines are shown in (G)) or in U2OS-delta-RE-TetR-GFP (n=2, each dot represents one experiment). Cells were transfected, or not, with I-SceI-GR-LBD and treated, or not, with TA for two hours. (G) Percentage of GFP-positive cells seven days after DSB in six independently established polyclonal cell lines and thirteen different monoclonal cell lines. GFP, in control (dark grey) or I-SceI induced (light grey) condition (error bars indicate the SEM for n≥3 independent experiments; dots represent individual values when n=1 or 2). (H) I-SceI-dependent increase in GFP-positive cells in cell line established without the LacO repeats component normalised to the original cell line (n=1).

A

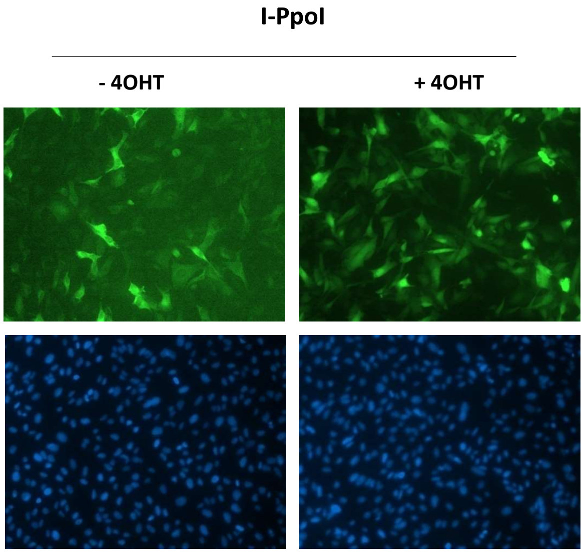

B

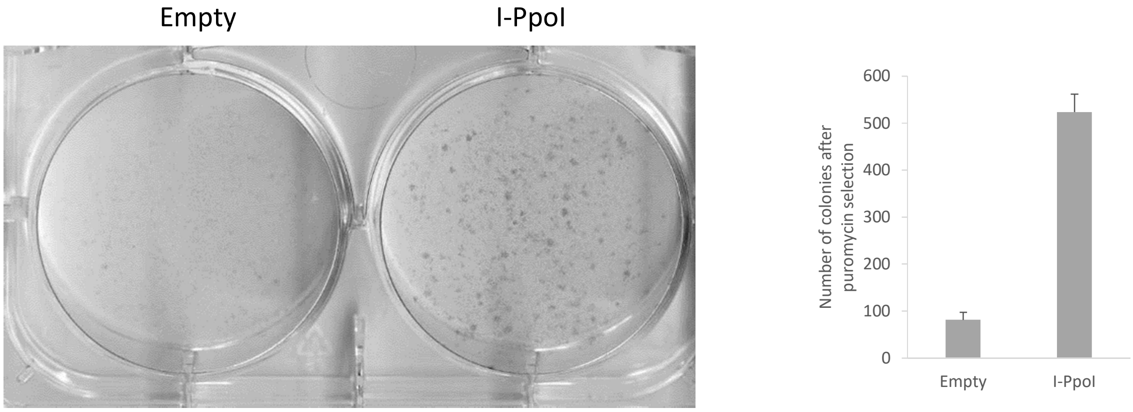

C

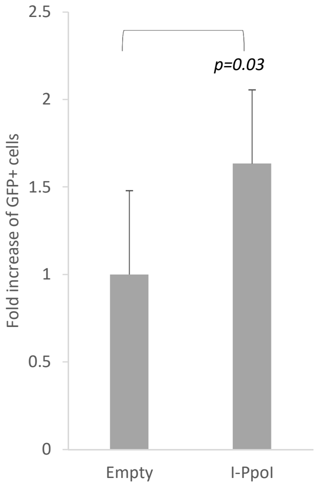

Figure S2: I-PpoI treatment:

(A) Immunofluorescence of cells infected with retrovirus coding for an inducible HA-ER-I-PpoI and treated for 24h with 4-hydroxytamoxifen (4OHT). I-PpoI is visualized with a specific anti-HA antibody (secondary FITC) and the nuclei are stained with DAPI. (B) Clonogenicity assay of cells treated with I-PpoI (+4OHT) compared to cell infected with an empty virus (+4OHT). Staining with brilliant blue (left panel) and ImageJ® quantification (right panel) of one experiment performed in triplicate (average and standard deviation are indicated). (C) FACS quantification of the fold increase of GFP-positive cells induced by I-PpoI seven days after damage induction, normalised to cells infected with empty virus (n=3).

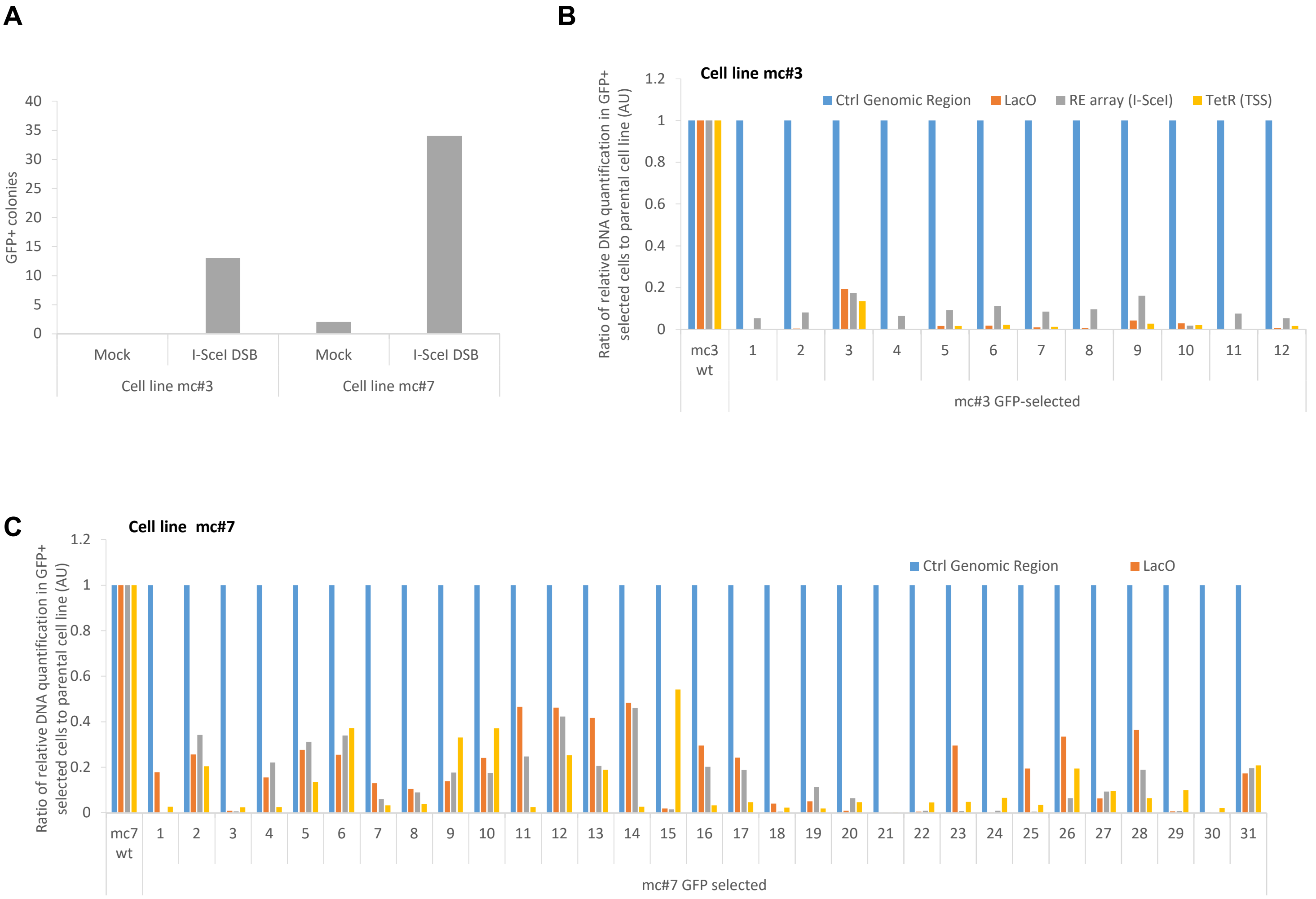

Figure S3: Analysis of GFP-positive clones after I-SceI induction:

(A) Histogram count of GFP-positives single clones selected after plating 3800 cells in 4 different 96-wells plates (10 cells per well). Clones were from two U2OS-RE-Tetr-GFP monoclonal cell lines, mc#3 and mc#7, with and without I-SceI induction. Wells containing single GFP-positive clones were then treated with puromycin to remove GFP-negative clones. (B-C) qPCR assay as described in figure 1F with specific primers for genomic control (blue), LacO repeat (orange), RE array (grey) and TetR gene (yellow), on genomic DNA extracted from clones isolated from cell line mc#3 (B) and from cell line mc#7 (C).

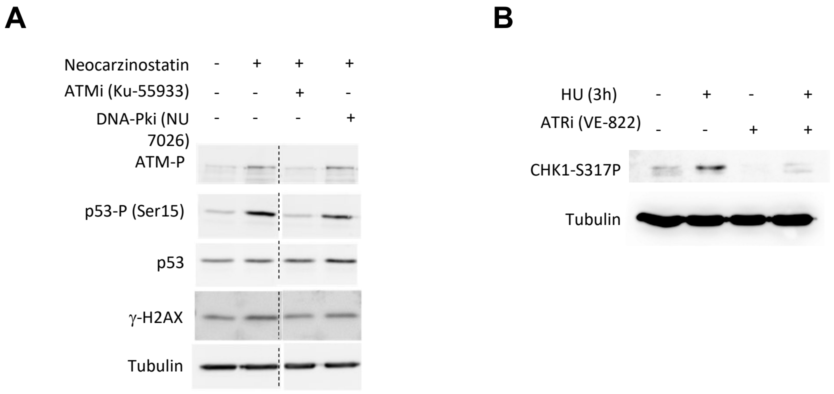

Figure S4: The DSB-induced deletions are independent of ATM, ATR and DNA-PK activation and DNA replication at the time of damage:

(A) Immunoblot of U2OS damaged with the radiomimetic drug neocarzinostatin (50 ng/ml for 30 minutes) without or with ATM (Ku-55933) or DNA-PK (Nu-7026) inhibitors. Specific antibodies against ATM-P, p53-P (Ser15), p53, γH2AX and tubulin were used. (B) Immunoblot of U2OS treated with hydroxyurea (HU) for 3h, without or with ATR inhibitors. Specific antibodies against CHK1-P (Ser317) and tubulin were used.

Figure S5. Manipulation of R-loop processing alters levels of DSB-induced deletions

(A) Analysis of transcript levels, assessed by RT-qPCR, after siRNA targeting Senataxin, XPF, TopI and XPG, normalised to GAPDH mRNA levels and siControl (n=3; error bars represent standard deviation). (B) Fold increase of GFP-positive cells normalised to the untreated control cells seven days after I-SceI induction in Senataxin-depleted cells (n=6). (C) Immunoblot of whole cell extract from cell expressing, or not, RNaseH1 and treated, or not, with CPT (5µM, 1h), using a specific antibody against RNaseH1 and phospho-p53 (S15). The asterisk (*) indicates a nonspecific band used as a loading control. (D) Effect of RNaseH1 overexpression on the GFP-positive population of cells with, and without, DSB: fold increase of GFP-positive cells in cells transfected with RNaseH1, or not, and co-transfected, or not, with I-SceI, seven days after DSB normalised to control cells without I-SceI (n=4). (E) Relative amount of TetR mRNA, measured by RT-qPCR, in control and RNaseH1-expressing cells (n=2, each dot represents one experiment). (F-J) Fold increase of GFP-positive cells normalised to the untreated control cells seven days after I-SceI induction in: (F) CPT-treated cells (n=7); (G) TopI-depleted cells (n=6); (H) XPF-depleted cells (n=6); (I) XPG-depleted cells (n=3) and (J) ERCC8-depleted cells (n=3).

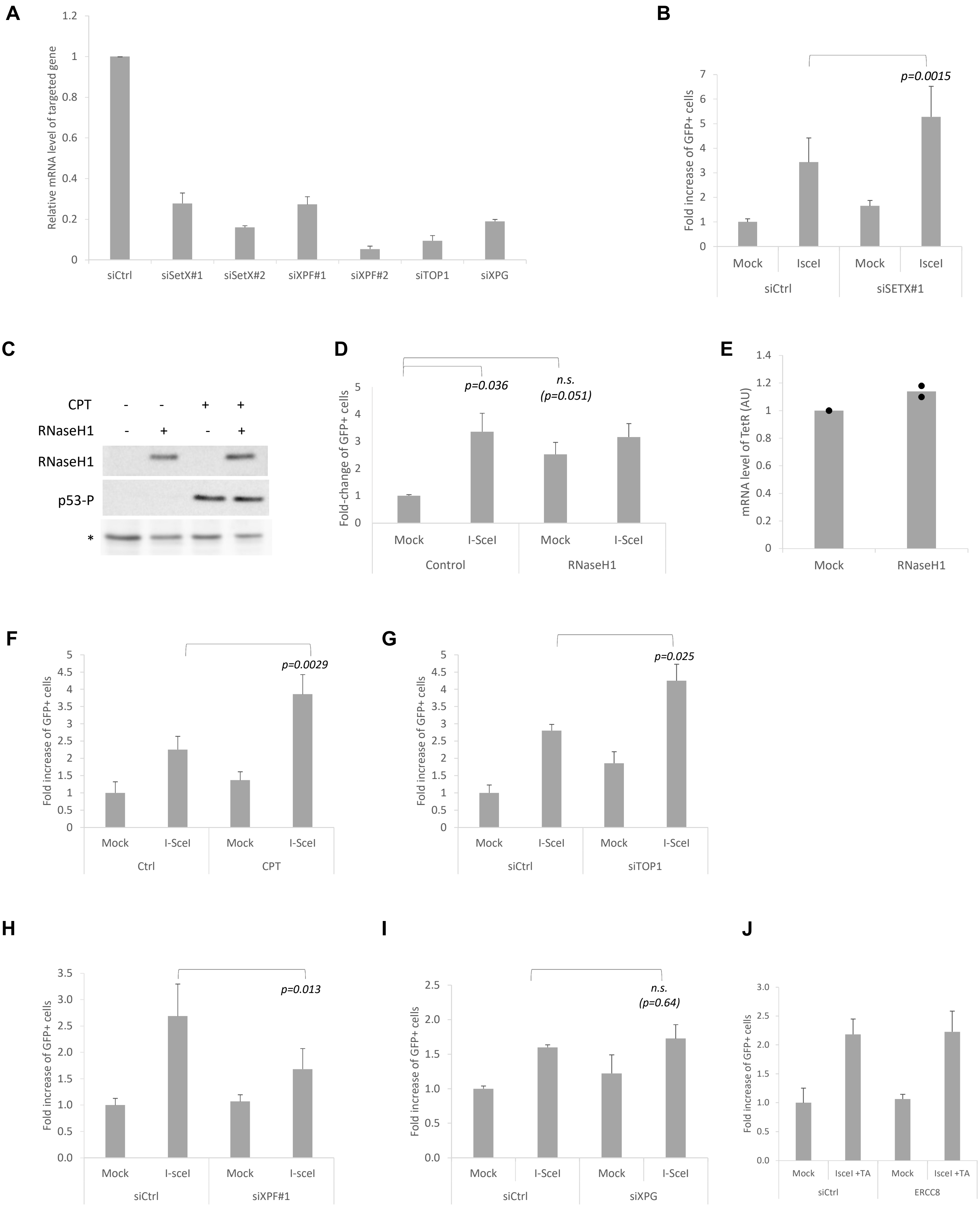

Figure S5. Manipulation of R-loop processing alters levels of DSB-induced deletions

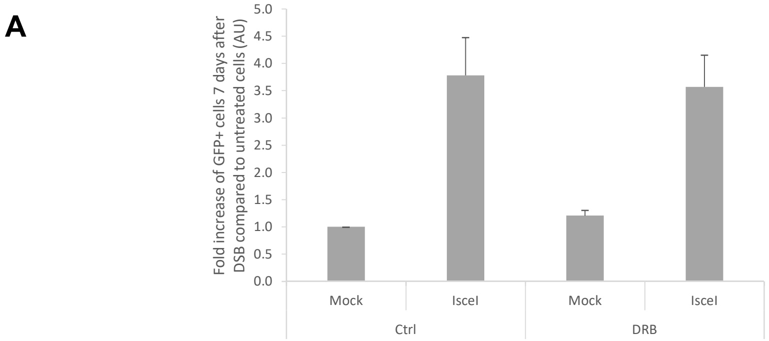

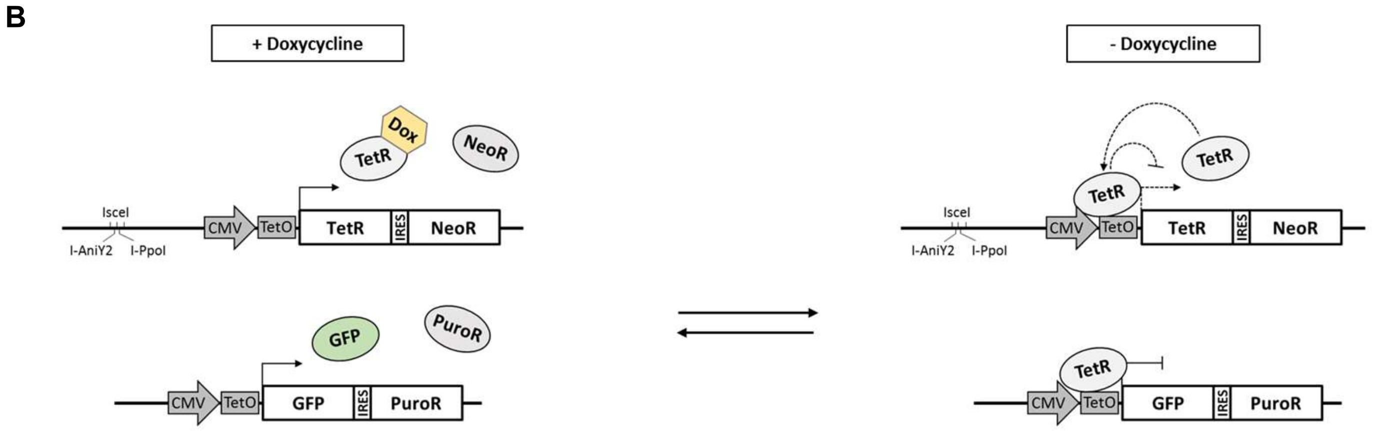

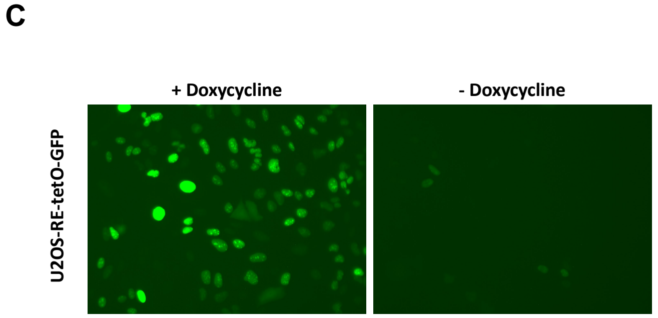

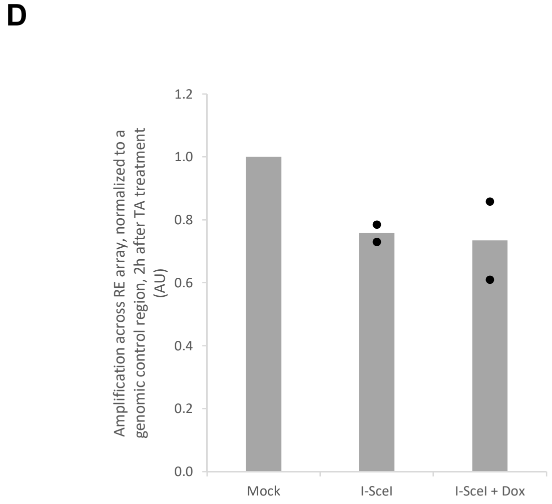

Figure S6. Characterisation of transcription activity regulation system:

(A) FACS analysis of the fold-increase of GFP-positive cells compared to the untreated control cells seven days after DSB in cells treated or not with the transcription inhibitor DRB (n=7). (B) Schematic representation of the transcription activity of TetR-IRES-NeoR and GFP-IRES-PuroR cassettes with (left panel) or without (right panel) doxycycline. (C) Fluorescence images of U2OS-RE-tetO-GFP cells growing in media with (left panel) or without (right panel) doxycycline. (D) Evaluation of cutting efficiency by I-SceI in low (I-SceI only) or in high (I-SceI + Dox) level of transcription, as described in Figure S1D (n=2, each dot represents one experiment).

Table 1 : List of siRNA used

Name	Dharmacon catalog number	Target sequence
siCtrl	D-001810-10-05	UGGUUUACAUGUCGACUAA
		UGGUUUACAUGUUGUGUGA
		UGGUUUACAUGUUUUCUGA
		UGGUUUACAUGUUUUCCUA
siSetX#1	L-021420-00	GCACGUCAGUCAUGCGUAA
		GCAAUAAGCUCAUCCUAGU
		GCUCAACUCUCCAAAUAGA
		UAGCACAGGUUGUUAAUCA
siSetX#2	D-021420-01-0002	GCACGUCAGUCAUGCGUAA
siXPF#1	L-019946-00	CCAAACAGCUUUAUGAUUU
		GCACCUCGAUGUUUAUAAA
		CGGAAGAAAUUAAGCAUGA
		UGACAAGGGUACUACAUGA
siXPF#2	D-019946-02-0002	GUAGGAUACUUGUGGUUGA
siMus81#1	L-016143-01	CAGCCCUGGUGGAUCGAUA
		CAUUAAGUGUGGGCGUCUA
		UGACCCACACGGUGCGCAA
		CUCAGGAGCCCGAGUGAUA
siMus81#2	D-016143-02-0002	GGGAGCACCUGAAUCCUAA
siTOP1	L-005278-00	GAAAAUGGCUUCUCUAGUC
		GAUUUCCGAUUGAAUGAUU
		GCACAUCAAUCUACACCCA
		CGAAGAAGGUAGUAGAGUC
siXPG	D-006626-01-0002	CAUGAAAUCUUGACUGAUA
ERCC8	L-011008-00	GUAAAGCAGUGUGUUCCAU
		CAGACAAUCUUAUUACACA
		CAUCAUAUGUCUCCAGUCU
		GAUUGUACUUUAUGACCUU

Table 2 : List of primers used

qPCR primers	Sequence 5'-->3'
TetR f	AGAGCATCAAGTCGCTAAAGAA
TetR r	TCTGCACCTTGGTGATCAAATA
GAPDH f	TGCACCACCAACTGCTTAGC
GAPDH r	GGCATGGACTGTGGTCATGAG
Genomic control #1 f	GTGCATCTGTCCACAGCAAAGGTT
Genomic control #1 r	AAGCTGGGTGGATGTGAATCCTGT
Genomic control #2 f	AGCACGTGCATGATTCTGTAGGGT
Genomic control #2 r	GAGTCAGGGTAACGGTCAAAGAAG
LacO f	TGTTATCCGCTCACAATTCCT
LacO r	GACAAGTAGCCTCTAGCATCAC
IsceI f	CCGCATAGTTAAGCCAGTATCT
IsceI r	GCCCGTACGACTAGTCAATAAT
TSS f	TGGATAGCGGTTTGACTCAC
TSS r	GTAAGCAGTGGGTTCTCTAGTT
NeoR f	GCTATCAGGACATAGCGTTGG
NeoR r	CCTCTACAAATGTGGTATGGCT
SETX#1F	TGACATCACTGGGCAAGACTT
SETX#1R	CCAACGCCGAACCATTTCATT
SETX#2F	GCCAGCGTTACACTGTTTTATG
SETX#2R	GTAGCTTGCGTTGTTGATAATGG
XPF#1F	GGAACTGCTCGACACTGACG
XPF#1R	GCGAGGGAGGTGTTCAACTC
XPF#2F	TCCCTCGCCGTGTAACAAATG
XPF#2R	GAGGCGCAAGATGAATGCTTC
TOP1#1F	TCGAAGCGGATTTCCGATTGA
TOP1#1R	CTTTGTGCCGGTGTTCTCGAT
TOP1#2F	AAGGTCCAGTATTTGCCCCAC
TOP1#2R	ATTCATGGTCGAGCATTTTTGC
XPG F	GATGGGGATGCTCCACTATTG
XPG R	ACTGGACGCTAAGTCCTTTCT
I-SceI cleavage f	GCATCATTACACCTGCAGCT
I-SceI cleavage r	ATTCCGCGGGAATTCGTTG
APOE (DRIP+) f	CCGGTGAGAAGCGCAGTCGG
APOE (DRIP+) r	CCCAAGCCCGACCCCGAGTA
Intergenic (DRIP-) f	CTGTACCTGGGGTTCATTCATT
Intergenic (DRIP-) r	CAGTAAGCCGTTCACTCTCAC
IsceI array (DRIP) f	CCGCATAGTTAAGCCAGTATCT
IsceI array (DRIP) r	GCCCGTACGACTAGTCAATAAT
